# On the preconditioned GAOR method for a linear complementarity problem with an *M*-matrix

**DOI:** 10.1186/s13660-018-1789-5

**Published:** 2018-07-27

**Authors:** Shu-Xin Miao, Dan Zhang

**Affiliations:** College of Mathematics and Statistics, Northwest Normal University, Lanzhou, People’s Republic of China

**Keywords:** 65F10, 65F15, Linear complementarity problem, Preconditioner, Preconditioned GAOR method, *M*-matrix

## Abstract

Recently, based on the Hadjidimos preconditioner, a preconditioned GAOR method was proposed for solving the linear complementarity problem (Liu and Li in East Asian J. Appl. Math. 2:94–107, 2012). In this paper, we propose a new preconditioned GAOR method for solving the linear complementarity problem with an *M*-matrix. The convergence of the proposed method is analyzed, and the comparison results are obtained to show it accelerates the convergence of the original GAOR method and the preconditioned GAOR method in (Liu and Li in East Asian J. Appl. Math. 2:94–107, 2012). Numerical examples verify the theoretical analysis.

## Introduction

For a given matrix $A\in \mathbb{R}^{n\times n}$ and a vector $f\in \mathbb{R}^{n}$, the linear complementarity problem, abbreviated as LCP, consists of finding a vector $x\in \mathbb{R}^{n}$ such that

1 $$ x\geq 0, \qquad r:=Ax-f\geq 0, \quad\text{and}\quad x^{T}r=0. $$

Here, the notation “≥” denotes the componentwise defined partial ordering between two vectors and the superscript *T* denotes the transpose of a vector.

The LCP of the form (1) arises in many scientific computing and engineering applications, for example, the Nash equilibrium point of a bimatrix game, the contact problem, and the free boundary problem for journal bearings; see [5, 16] and the references therein. As is known, LCP (1) possesses a unique solution if and only if $A\in \mathbb{R}^{n\times n}$ is a *P*-matrix, namely a matrix whose all principal submatrices have positive determinants; see [4, 5, 16]. A matrix *A* is called an *M*-matrix if its inverse is nonnegative and all its off-diagonal entries are nonpositive. A positive diagonal *M*-matrix is a *P*-matrix, and LCP (1) with an *M*-matrix has the unique solution [3].

Because of the wide applications, the research on the numerical methods for solving (1) has attracted much attention. There are some iterative methods for obtaining the solution of the LCP, including the projected methods [8, 9, 12], the modulus algorithms [10], and the modulus-based matrix splitting iterative methods [2, 6, 18, 19], see [9] for a survey of the iterative method for LCP (1). We consider the generalized AOR (GAOR) method [8, 12], which is a special case of the projected method, for solving LCP (1) with an *M*-matrix. For accelerating the convergence rate of the GAOR method, the preconditioned GAOR method is proposed in [13], based on the preconditioner in [7], for LCP (1) with an *M*-matrix. In this paper, a new preconditioner is proposed to accelerate the convergence rate of the GAOR method for solving LCP (1).

The outline of the rest of the paper is as follows. In Sect. 2, some preliminaries about the projected method are reviewed, and the new preconditioner for preconditioned GAOR method is introduced. Convergence analysis is given in Sect. 3. The convergence rates of the proposed preconditioned GAOR method are compared with the convergence rates of the preconditioned GAOR method in [13] and the convergence rates of the original GAOR method for LCP with an *M*-matrix in Sect. 4, which shows that the proposed preconditioned GAOR converges faster than the preconditioned GAOR method in [13] and the original GAOR method. Numerical examples are given to verify the theoretical results in Sect. 5. Finally, conclusions are drawn in Sect. 6.

## Preliminaries

We give some of the notations, definitions, and lemmas which will be used in the sequel. For $A=(a_{i,j})$, $B=(b_{i,j})\in R^{n\times n}$, we write $A\geq B$ if $a_{i,j}\geq b_{i,j}$ holds for all $i,j=1,2, \ldots ,n$. $A\geq O$ is called nonnegative if $a_{i,j}\geq 0$ for all $i,j=1,2,\ldots ,n$, where *O* is an $n\times n$ zero matrix. For the vectors $a, b\in R^{n\times 1}$, $a\geq b$ and $a\geq 0$ can be defined in a similar manner. By $|A|=(|a_{ij}|)$ we define the absolute value of a given matrix $A\in \mathbb{R}^{n\times n}$. We denote the $n\times n$ diagonal matrix coinciding in its diagonal with matrix $C\in \mathbb{R}^{n\times n}$ by $\operatorname{diag}(C)$. For simplicity, we may assume that $a_{ii}=1$ ($i=1,2 \dots n $).

### Lemma 1

([17])

*Let* $A = [a_{i j}] \in R^{n \times n}$ *and* $a_{i j} \leq 0$ *for* $i\neq j$. *A* *is an* *M*-*matrix if and only if there exists a positive vector* *y* *such that* $Ay > 0$.

There are equivalent conditions of an *M*-matrix, a nonsingular *M*-matrix is a monotone matrix, see [4].

### Definition 1

([4])

For a matrix $A\in \mathbb{R}^{n\times n}$, the representation $A=M-N$ is called a splitting of *A* if *M* is nonsingular. Then $A=M-N$ is called weak regular if $M^{-1}\geq 0$ and $M^{-1}N\geq 0$.

For the weak regular splittings of different monotone matrices, there is a comparison result as follows.

### Lemma 2

([4])

*Suppose that* $A_{1}=M_{1}-N_{1}$ *and* $A_{2}=M _{2}-N_{2}$ *are weak regular splittings of the monotone matrices* $A_{1}$ *and* $A_{2}$, *respectively*, *such that* $M_{1}^{-1}\leq M_{2} ^{-1}$. *If* $M_{1}^{-1}N_{1}$ *has a positive Perron vector*, *then*

$$ \rho \bigl(M_{2}^{-1}N_{2} \bigr)\leq \rho \bigl(M_{1}^{-1}N_{1} \bigr). $$

The following lemma is taken from [11].

### Lemma 3

([11])

*Let* *A* *be an* *M*-*matrix and* *x* *be a solution of LCP* (1). *If* $f_{i}>0$, *then* $x_{i}>0$ *and therefore* $\sum^{n}_{j=1} a_{ij}x_{j}-f_{i}=0$. *Moreover*, *if* $f\leq 0$, *then* $x=0$ *is the solution of LCP* (1).

For the study of the projected methods, the following definition is needed.

### Definition 2

([15])

Given any vector $x\in \mathbb{R}^{n}$, $x_{+}$ denotes the vector with components

$$ (x_{+})_{i}=\max \{x_{i}, 0\} \quad \forall i \in N:=\{1, 2,\ldots , n\}. $$

From Definition 2, the following properties hold for any $x, y\in \mathbb{R}^{n}$:

(i) $(x+y)_{+}\leq x_{+}+y_{+}$;
(ii) $x_{+}-y_{+}\leq (x-y)_{+}$;
(iii) $|x|=x_{+}+(-x)_{+}$;
(iv) $x\leq y\Rightarrow x_{+}\leq y_{+}$.

Using Definition 2, LCP (1) is analogous to [1]

2 $$ x= \bigl( x-D^{-1}(Ax-f) \bigr) _{+}, $$

where $D=\operatorname{diag}(A)$ is a nonsingular matrix. From (2) and the splitting of *A* as $A=D-L-U$, here *D*, −*L*, −*U* are the diagonal, the strictly lower, and the strictly upper triangular parts of A, respectively. The GAOR method for solving (1) can be defined as (see [8, 12])

3 $$ x^{k+1}= \bigl( x^{k}-D^{-1} \bigl[ \alpha \Omega L x^{k+1}+(\Omega A-\alpha \Omega L)x^{k}-\Omega f \bigr] \bigr) _{+}, \quad k=0, 1,\ldots , $$

where $\Omega =\operatorname{diag}(\omega_{1}, \omega_{2},\ldots ,\omega_{n})$ is a positive diagonal matrix and *α* is a positive real number. Let $J=D^{-1}(L+U)$, $G=I-\alpha \Omega D^{-1}|L|$, and $F=|I-D^{-1}( \Omega A-\alpha \Omega L)|$, the following result concerns the convergence of the GAOR method for solving LCP (1).

### Lemma 4

([12])

*Suppose that* *A* *is a positive diagonal* *H*-*matrix*. *Then*, *for any initial vector* $x^{0}\in R^{n}$, *the iterative sequence* $\{x^{k} \}$ *generated by the GAOR method* (3) *converges to the unique solution* $x^{*}$ *of* $LCP$ (1), *and*

$$ \rho \bigl(G^{-1}F \bigr) \leq \max_{1\leq i \leq n} \bigl\{ \vert 1 - \omega_{i} \vert + \omega _{i}\rho \bigl( \vert J \vert \bigr) \bigr\} < 1 , $$

*where* $0 < \omega_{i} < 2/[1 + \rho (|J|)]$ *and* $0 \leq \alpha \leq 1 $.

For accelerating the convergence rate of the GAOR method, a preconditioner, based on the Hadjidimos preconditioner [7], is proposed in [13]

$$ \widetilde{P}=\left [ \begin{matrix} 1 & & \\ -\gamma_{2} a_{21}-\beta_{2} & 1 & \\ \vdots & & & & \ddots & \\ -\gamma_{i} a_{i1}-\beta_{i} & & & & & & 1 & \\ \vdots & & & & & & & \ddots \\ -\gamma_{n} a_{n1}-\beta_{n} & & & & & & & & 1 \end{matrix} \right ] , $$

where $\gamma_{i}\geq 0$ ($i=2,\ldots ,n$) and $\beta_{i}$ ($i=2,\ldots ,n$) are constants. It has been showed that the preconditioned matrix $\widetilde{A}=\widetilde{P}A$ is also an *M*-matrix when *A* is an *M*-matrix [13], hence the equivalent linear complementarity problem of LCP (1)

$$ x\geq 0, \qquad \tilde{r}= \tilde{A}x-\tilde{P}f\geq 0, \qquad x^{T} \tilde{r}=0 $$

has the unique solution [3]. The preconditioned GAOR method for solving LCP (1) is defined [13] as

4 $$ x^{k+1}= \bigl( x^{k}-\widetilde{D}^{-1} \bigl[\alpha \Omega \widetilde{L} x ^{k+1}+(\Omega \widetilde{A}-\alpha \Omega \widetilde{L})x^{k}-\Omega \widetilde{P}f \bigr] \bigr) _{+}, \quad k=0, 1,\ldots , $$

based on the splitting $\widetilde{A}=\widetilde{D}-\widetilde{L}- \widetilde{U}$, please refer to [13] for more details.

Note that the preconditioning effect of *P̃* is not observed on the first row of matrix *A*. In order to provide the preconditioning effect on all the rows of *A*, in this paper, we propose the following preconditioner:

5 $$ \widehat{P}=\left [ \begin{matrix} 1 & &&&&&&& -\gamma_{1} a_{1n}-\beta_{1} \\ -\gamma_{2} a_{21}-\beta_{2} & 1 & \\ \vdots & & & & \ddots & \\ -\gamma_{i} a_{i1}-\beta_{i} & & & & & & 1 & \\ \vdots & & & & & & & \ddots \\ -\gamma_{n} a_{n1}-\beta_{n} & & & & & & & & 1 \end{matrix} \right ] , $$

where $\gamma_{i}\geq 0$ ($i=1,\ldots ,n$) and $\beta_{i}$ ($i=1,\ldots ,n$) are constants.

Let the preconditioned matrix $\widehat{A}=\widehat{P}A$ be split as $\widehat{A}=\widehat{D}-\widehat{L}-\widehat{U}$, where *D̂*, *L̂*, and *Û* are the diagonal, lower triangular, and upper triangular matrices, then the preconditioner GAOR method for solving LCP (1) is defined as

6 $$ x^{k+1}= \bigl( x^{k}-\widehat{D}^{-1} \bigl[\alpha \Omega \widehat{L} x^{k+1}+( \Omega \widehat{A}-\alpha \Omega \widehat{L})x^{k}-\Omega \widehat{P}f \bigr] \bigr) _{+}, \quad k=0, 1,\ldots . $$

## Convergence analysis

In this section, we will consider the convergence of the preconditioned GAOR method (6) for solving LCP (1). In what follows, we make the assumptions:

(H0): $f_{1}>0$ and $f_{n}>0$;
(H1): $0\leq \gamma_{i}\leq 1$ for $i=1,2,\ldots , n$;
(H2): $-\gamma_{1} a_{1n}+a_{1n}\leq \beta_{1}\leq -\gamma_{1} a_{1n}$;
(H3): $-\gamma_{i} a_{i1}+a_{i1}\leq \beta_{i}\leq -\gamma_{i} a_{i1} $ for $i=2,3,\ldots ,n$.

### Theorem 1

*Let* $\widehat{A}=\widehat{P}A=[\hat{a}_{ij}]$ *and* $\hat{f}= \widehat{P}f=[\hat{f}_{i}]$. *If* (H0)–(H3) *are satisfied*, *then LCP* (1) *is equivalent to the linear complementarity problem*

7 $$ x\geq 0, \qquad \hat{r}=\hat{A}x-\hat{f}\geq 0, \qquad x^{T}\hat{r}=0. $$

### Proof

After some calculations, the elements of *Â* and *f̂* can be expressed, respectively, as follows:

8 $$ \hat{a}_{ij}= \textstyle\begin{cases} a_{1j}-(\gamma_{1}a_{1n}+\beta_{1})a_{nj},&i=1,j=1,2,\ldots ,n, \\ a_{ij}-(\gamma_{i}a_{i1}+\beta_{i})a_{1j},& i\neq 1,j=1,2,\ldots ,n, \end{cases} $$

and

9 $$ \hat{f}_{i}= \textstyle\begin{cases} f_{1}-(\gamma_{1}a_{1n}+\beta_{1})f_{n},&i=1, \\ f_{i}-(\gamma_{i}a_{i1}+\beta_{i})f_{1},&i\neq 1. \end{cases} $$

Let *x* be a solution of LCP (1). Since $f_{1}>0$ and $f_{n}>0$, from Lemma 3 we have $x_{1}>0$, $\sum^{n}_{j=1}a _{1j}x_{j}-f_{1}=0$, and $x_{n}>0$, $\sum^{n}_{j=1}a_{nj}x_{j}-f_{n}=0$. Therefore, on the one hand, if $i=1$, then

10 $$ \begin{aligned}[b] \sum_{j=1}^{n} \hat{a}_{ij}x_{j}-\hat{f}_{i}&=\sum _{j=1}^{n} \bigl( a _{1j}-( \gamma_{1}a_{1n}+\beta_{1})a_{nj} \bigr) x_{j}- \bigl( f_{1}-( \gamma_{1}a_{1n}+ \beta_{1})f_{n} \bigr) \\ &=\sum_{j=1}^{n} a_{1j}x_{j}-f_{1} -(\gamma_{1}a_{1n}+\beta_{1}) \Biggl( \sum _{j=1}^{n}a_{nj}x_{j}-f_{n} \Biggr) \\ &=\sum_{j=1}^{n} a_{1j}x_{j}-f_{1}. \end{aligned} $$

On the other hand, if $i\neq 1$, then

11 $$ \begin{aligned}[b] \sum_{j=1}^{n} \hat{a}_{ij}x_{j}-\hat{f}_{i}&=\sum _{j=1}^{n} \bigl( a _{ij}-( \gamma_{i}a_{i1}+\beta_{i})a_{1j} \bigr) x_{j}- \bigl( f_{i}-( \gamma_{i}a_{i1}+ \beta_{i})f_{1} \bigr) \\ &=\sum_{j=1}^{n} a_{ij}x_{j}-f_{i} -(\gamma_{i}a_{i1}+\beta_{i}) \Biggl( \sum _{j=1}^{n}a_{1j}x_{j}-f_{1} \Biggr) \\ &=\sum_{j=1}^{n} a_{ij}x_{j}-f_{i}. \end{aligned} $$

Thus, *x* is a solution of LCP (7).

Conversely, suppose that *x* is the solution to LCP (7), assumptions (H0), (H3) and (9) imply that $\beta_{n} +\gamma_{n} a_{n1} \leq 0 $, so $-(\beta_{n} +\gamma_{n} a _{n1})f_{1} \geq 0$, then $f_{n}-(\beta_{n} +\gamma_{n} a_{n1})f_{1} \geq 0$, we get that $\hat{f}_{n}>0$. Similarly, from assumptions (H0), (H2) and (9), we can obtain $\hat{f}_{1}>0$. It follows from Lemma 3 that $x_{1}>0$, $\sum^{n}_{j=1}\hat{a}_{1j}x_{j}- \hat{f}_{1}=0$, and $x_{n}>0$, $\sum^{n}_{j=1}\hat{a}_{nj}x_{j}- \hat{f}_{n}=0$. This together with (10) and (11) gives $\sum^{n}_{j=1}a_{ij}x_{j}-f_{i}=0$. Thus, for $i=1$, we have

$$ \begin{aligned}[b] \sum_{j=1}^{n} ( a_{ij}x_{j}-f_{i} ) &=\sum _{j=1}^{n} ( a _{ij}x_{j}-f_{i} ) -(\gamma_{1}a_{1n}+\beta_{1}) \Biggl( \sum _{j=1} ^{n}a_{nj}x_{j}-f_{n} \Biggr) \\ &=\sum_{j=1}^{n} \bigl( a_{1j}-( \gamma_{1}a_{1n}+\beta_{1})a_{nj} \bigr) x _{j}- \bigl( f_{1}-(\gamma_{1}a_{1n}+ \beta_{1})f_{n} \bigr) \\ &=\sum_{j=1}^{n}\hat{a}_{ij}x_{j}- \hat{f}_{i}. \end{aligned} $$

While for $i\neq 1$, we can deduce that

$$ \begin{aligned} \sum_{j=1}^{n} ( a_{ij}x_{j}-f_{i} ) &=\sum _{j=1}^{n} ( a _{ij}x_{j}-f_{i} ) -(\gamma_{i}a_{in}+\beta_{i}) \Biggl( \sum _{j=1} ^{n}a_{1j}x_{j}-f_{1} \Biggr) \\ &=\sum_{j=1}^{n} \bigl( a_{ij}-( \gamma_{i}a_{i1}+\beta_{i})a_{1j} \bigr) x _{j}- \bigl( f_{i}-(\gamma_{i}a_{i1}+ \beta_{i})f_{1} \bigr) \\ &=\sum_{j=1}^{n}\hat{a}_{ij}x_{j}- \hat{f}_{i}. \end{aligned} $$

Hence, *x* is the solution of LCP (1). □

### Lemma 5

*If* *A* *is an* *M*-*matrix*, (H1)–(H3) *hold*, *then* $\widehat{A}= \widehat{P}A$ *is an* *M*-*matrix*.

### Proof

If *A* is an *M*-matrix, then $a_{ij}<0$ for $i\neq j$ and $a_{1i}a_{i1}<1$, which leads to $a_{1i}>1/a_{i1}$. Otherwise, from (8) and assumption (H2), $-\gamma_{1} a_{1n}+a _{1n}\leq \beta_{1}\leq -\gamma_{1} a_{1n}$, we have that $\beta_{1} + \gamma_{1} a_{1n}\leq 0$ and $\beta_{1} +\gamma_{1} a_{1n}\geq a_{1n}> 1/a_{n1}$. Then $\hat{a}_{11}= a_{11}-(\beta_{1} +\gamma_{1} a_{1n})a _{n1} > 1-1/a_{n1}\ast a_{n1}=0 $.

Similarly, we can obtain

$$ \begin{gathered} \hat{a}_{11}=a_{11}-( \gamma_{1}a_{1n}+\beta_{1})a_{n1}>0, \\ \hat{a}_{ii}=a_{ii}-(\gamma_{i}a_{i1}+ \beta_{i})a_{1i}>0,\quad i\neq 1, \\ \hat{a}_{1j}=a_{1j}-(\gamma_{1}a_{1n}+ \beta_{1})a_{nj}< 0,\quad \\ \hat{a}_{ij}=a_{ij}-(\gamma_{i}a_{i1}+ \beta_{i})a_{1j}< 0,\quad i\neq 1,j. \end{gathered} $$

From Lemma 1 there exists a positive vector $y > 0$ such that $Ay > 0$. Note that $\widehat{P}>0$, thus $\widehat{A} y = \widehat{P}Ay > 0$, and from Lemma 1, *Â* is an *M*-matrix. □

### Theorem 2

*Let* *A* *be a diagonally dominant* *M*-*matrix*. *If* (H0)–(H3) *hold*, *then for* $0\leq \omega_{i}\leq 2/[1+\rho (|\hat{J}|)]$ ($i=1,\ldots ,n$) *and* $0\leq \alpha \leq 1$, *the iterative sequence of the preconditioned GAOR method* (6) *converges to the unique solution* $x^{\ast }$ *of LCP* (1), *here* $\hat{J}=\widehat{D}^{-1}(\widehat{L}+\widehat{U})$.

### Proof

From Lemma 5, we know that *Â* is a diagonally dominant *H*-matrix. Thus from Theorem 1 and Lemma 4, the preconditioned GAOR method (6) converges to the unique solution of LCP (1). □

## Comparison results

In this section, we will compare the convergence rate of the preconditioned GAOR method (6) with that of the GAOR method (3) and that of the preconditioned GAOR method (4) [13]. For simplicity, we may assume that $a_{ii}=1$, $i=1,\ldots ,n$. For this case, $G=I-\alpha \Omega |L|$, $F=|I-(\Omega A-\alpha \Omega L)|$, and *D̃*, *L̃*, and *Ũ* can be expressed as $\widetilde{D}=I-S_{D}$, $\widetilde{L}=L-S+S_{L}$, $\widetilde{U}=U+S_{U}$ with

$$\begin{aligned}& S=\left [ \begin{matrix} 0 & & \\ -\gamma_{2} a_{21}-\beta_{2} & 0 & \\ \vdots & & & & \ddots & \\ -\gamma_{i} a_{i1}-\beta_{i} & & & & & & 0 & \\ \vdots & & & & & & & \ddots \\ -\gamma_{n} a_{n1}-\beta_{n} & & & & & & & & 0 \end{matrix} \right ] , \\& S_{D}=\left [ \begin{matrix} 0 & & \\ & (\gamma_{2} a_{21}+\beta_{2})a_{12} & \\ & & (\gamma_{3} a_{31}+\beta_{3})a_{13}& & \\ & & & \ddots & \\ & & & & (\gamma_{n} a_{n1}+\beta_{n})a_{1n} \end{matrix} \right ] , \\& S_{L}=\left [ \begin{matrix} 0 & 0&\cdots &0&0 \\ 0 & 0&\cdots &0&0 \\ 0 & (\gamma_{3} a_{31}+\beta_{3})a_{12} & \cdots & 0 & 0 \\ \vdots &\vdots &\ddots &\vdots & \vdots \\ 0&(\gamma_{n} a_{n1}+\beta_{n})a_{12} &\cdots &(\gamma_{n} a_{n1}+ \beta_{n})a_{1,n-1} & 0 \end{matrix} \right ] , \\& S_{U}=\left [ \begin{matrix} 0 & 0&\cdots &0&0 \\ 0 & 0&(\gamma_{2} a_{21}+\beta_{2})a_{13}&\cdots &(\gamma_{2} a_{21}+ \beta_{2})a_{1n} \\ \vdots &\vdots &\ddots &\vdots & \vdots \\ 0 & 0 & 0 & \cdots & (\gamma_{n-1} a_{n-1,1}+\beta_{n-1})a_{1n} \\ 0&0&0&\cdots &0 \end{matrix} \right ] . \end{aligned}$$

Moreover, the *D̂*, *L̂*, and *Û* can be expressed as $\widehat{D}=I-S_{D}-R_{D}$, $\widehat{L}=L-S+S_{L}$, $\widehat{U}=U+S_{U}-R+R_{U}$ with

$$ \begin{gathered} R=\left [ \begin{matrix} 0 & \cdots & 0&-\gamma_{1} a_{1n}-\beta_{1} \\ 0 & \cdots & 0&0 \\ \vdots &\ddots &\vdots &\vdots \\ 0& \cdots &0 &0 \end{matrix} \right ] , \\ R_{D}=\left [ \begin{matrix} (\gamma_{1} a_{1n}+\beta_{1})a_{n1} & & & \\ & 0 & & \\ &&\ddots & \\ & & &0 \end{matrix} \right ] , \\ R_{U}=\left [ \begin{matrix} 0 & (\gamma_{1} a_{1n}+\beta_{1})a_{n2}&\cdots &(\gamma_{1} a_{1n}+ \beta_{1})a_{nn} \\ 0 & 0&\cdots &0 \\ \vdots &\vdots &\ddots &\vdots \\ 0 & 0 & \cdots & 0 \end{matrix} \right ] . \end{gathered} $$

### Lemma 6

*If* *A* *is an* *M*-*matrix with diagonal elements* 1, (H1)–(H3) *hold*, *then* $0\leq \widehat{D}\leq \widetilde{D}$.

### Proof

Note that

$$ \widetilde{D}=\operatorname{diag} \bigl(1, 1-(\gamma_{2} a_{21}+\beta_{2})a_{12}, \ldots ,1-( \gamma_{n} a_{n1}+\beta_{n})a_{1n} \bigr) $$

and

$$ \widehat{D}=\operatorname{diag} \bigl(1-(\gamma_{1} a_{1n}+ \beta_{1})a_{n1}, 1-( \gamma_{2} a_{21}+ \beta_{2})a_{12},\ldots ,1-( \gamma_{n} a_{n1}+\beta _{n})a_{1n} \bigr). $$

Since *A* is an *M*-matrix with diagonal elements 1, we have $0< a_{ij}a_{ji}<1$ ($i\neq j$) [7]. Then assumptions (H1)–(H3) imply that

$$ 0< 1-(\gamma_{1} a_{1n}+\beta_{1})a_{n1}< 1 $$

and

$$ 0< 1-(\gamma_{i} a_{i1}+\beta_{i})a_{1i}< 1, \quad i=2,\ldots ,n. $$

Hence, $0\leq \widehat{D}\leq \widetilde{D}$. □

From Lemma 6 and the fact that $\widehat{L}=\widetilde{L}$, we have

12 $$ \widetilde{D}^{-1} \vert \widetilde{L} \vert \leq \widehat{D}^{-1} \vert \widehat{L} \vert . $$

Moreover, it is easy to check that the inequality

13 $$ \vert L \vert \leq \widetilde{D}^{-1} \vert \widetilde{L} \vert $$

holds [14]. Let $\widetilde{G}=I-\alpha \Omega \widetilde{D} ^{-1}|\widetilde{L}|$, $\widetilde{F}=|I-\widetilde{D}^{-1}(\Omega \widetilde{A}-\alpha \Omega \widetilde{L})|$, $\widehat{G}=I-\alpha \Omega \widehat{D}^{-1}|\widehat{L}|$, and $\widehat{F}=|I- \widehat{D}^{-1}(\Omega \widehat{A}-\alpha \Omega \widehat{L})|$. If (H1) and (H3) hold, then [14]

14 $$ \rho \bigl(\widetilde{G}^{-1}\widetilde{F} \bigr)\leq \rho \bigl(G^{-1}F \bigr)< 1. $$

The following theorem gives a comparison result between $\rho ( \widetilde{G}^{-1}\widetilde{F})$ with $\rho (\widehat{G}^{-1} \widehat{F})$.

### Theorem 3

*If* *A* *is an irreducible nonsingular* *M*-*matrix*, *and* (H0)–(H3) *hold*, *then*

$$ \rho \bigl(\widehat{G}^{-1}\widehat{F} \bigr)\leq \rho \bigl( \widetilde{G}^{-1} \widetilde{F} \bigr)< 1. $$

### Proof

It follows from Theorem 2 and (14) that $\rho (\widehat{G}^{-1}\widehat{F})<1$ and $\rho (\widetilde{G}^{-1} \widetilde{F})<1$, so we only need to show

$$ \rho \bigl(\widehat{G}^{-1}\widehat{F} \bigr)\leq \rho \bigl( \widetilde{G}^{-1} \widetilde{F} \bigr). $$

In terms of (12), we have

$$ I-\alpha \Omega \widehat{D}^{-1} \vert \widehat{L} \vert \leq I- \alpha \Omega \widetilde{D}^{-1} \vert \widetilde{L} \vert , $$

that is, $\widehat{G}\leq \widetilde{G}$. Note that *Ĝ* and *G̃* are *M*-matrices, hence

15 $$ 0\leq \widetilde{G}^{-1}\leq \widehat{G}^{-1}. $$

As $\widetilde{F}=|I-\widetilde{D}^{-1}(\Omega \widetilde{A}-\alpha \Omega \widetilde{L})|$ and $\widehat{F}=|I-\widehat{D}^{-1}(\Omega \widehat{A}-\alpha \Omega \widehat{L})|$ are nonnegative matrices, thus, this together with (15) yields $\widetilde{G}-\widetilde{F}$ and $\widehat{G}-\widehat{F}$ are the regular splitting of different monotone matrices $\widetilde{G}-\widetilde{F}$ and $\widehat{G}- \widehat{F}$, respectively. Moreover, for a nonnegative matrix $\widetilde{G}^{-1}\widetilde{F}$, according to Perron–Frobenius theorem (see [4]), there is a positive Perron vector *z* such that

$$ \widetilde{G}^{-1}\widetilde{F}z=\rho \bigl(\widetilde{G}^{-1} \widetilde{F} \bigr)z. $$

Hence, it follows from Lemma 2 that

$$ \rho \bigl(\widehat{G}^{-1}\widehat{F} \bigr)\leq \rho \bigl( \widetilde{G}^{-1} \widetilde{F} \bigr). $$

The proof is completed. □

### Remark 1

From Theorem 3 and (14), we can see that under assumptions (H0)–(H3), the inequalities

$$ \rho \bigl(\widehat{G}^{-1}\widehat{F} \bigr)\leq \rho \bigl( \widetilde{G}^{-1} \widetilde{F} \bigr)\leq \rho \bigl(G^{-1}F \bigr)< 1 $$

hold. This confirms that the proposed preconditioner *P̂* in (5) is more efficient than the preconditioner *P̃* [13] for accelerating the convergence rate of GAOR method for solving LCP (1) with an *M*-matrix.

## Numerical example

In this section, two examples are given for verifying the theoretical result.

### Example 1

Linear complementarity problem with coefficient matrix

$$ A_{1}=\left [ \begin{matrix} 1.00000 & -0.00580 & -0.19350 & -0.25471 & -0.03885 \\ -0.28424 & 1.00000 & -0.16748 & -0.21780 & -0.21577 \\ -0.24764 & -0.26973 & 1.00000 & -0.18723 & -0.08949 \\ -0.13880 & -0.01165 & -0.25120 & 1.00000 & -0.13236 \\ -0.25809 & -0.08162 & -0.13940 & -0.04890 & 1.00000 \end{matrix} \right ] . $$

Tables 1 and 2 list $\rho (G^{-1}F)$, $\rho (\widetilde{G}^{-1} \widetilde{F})$, and $\rho (\widehat{G}^{-1}\widehat{F})$ for different *α* and Ω.

**Table 1:** $\rho (G^{-1}F)$, $\rho (\widetilde{G}^{-1}\widetilde{F})$, and $\rho (\widehat{G}^{-1}\widehat{F})$ with $\alpha =0.1$ and $\omega_{i}=0.1$

Preconditioner	$(\gamma _{1},\ldots ,\gamma _{5})^{T}$	$(\beta _{1},\ldots ,\beta _{5})^{T}$	*ρ*(⋅)
*I*	–	–	0.96934
*P̃*	$(0,1,1,1,0.1)^{T}$	$(0,0.1,0,0.01,0.05)^{T}$	0.96311
	$(0,1,0,1,0)^{T}$	$(0,0,0.04,0.04,0.05)^{T}$	0.96750
*P̂*	$(1,1,1,1,0.1)^{T}$	$(0.03,0.1,0,0.01,0.05)^{T}$	**0.96292**
	$(1,1,0,1,0)^{T}$	$(0,0,0.04,0.04,0.05)^{T}$	0.96708

**Table 2:** $\rho (G^{-1}F)$, $\rho (\widetilde{G}^{-1}\widetilde{F})$, and $\rho (\widehat{G}^{-1}\widehat{F})$ with $\alpha =0.1$ and $\omega_{i}=0.9$

Preconditioner	$(\gamma _{1},\ldots ,\gamma _{5})^{T}$	$(\beta _{1},\ldots ,\beta _{5})^{T}$	*ρ*(⋅)
*I*	–	–	0.71690
*P̃*	$(0,1,1,1,0.1)^{T}$	$(0,0.1,0,0.01,0.05)^{T}$	0.67388
	$(0,1,0,1,0)^{T}$	$(0,0,0.04,0.04,0.05)^{T}$	0.70036
*P̂*	$(1,1,1,1,0.1)^{T}$	$(0.03,0.1,0,0.01,0.05)^{T}$	**0.67153**
	$(1,1,0,1,0)^{T}$	$(0,0,0.04,0.04,0.05)^{T}$	0.69654

### Example 2

Let the coefficient matrix *A* of LCP (1) be given by

$$ A=\left ( \begin{matrix} I-Q&U \\ L&I-R \end{matrix} \right ) , $$

where $Q=(q_{ij})\in R^{p\times p}$, $R=(r_{ij})\in R^{q\times q}$, $L=(l_{ij})\in R^{q\times p}$, and $U=(u_{ij})\in R^{p\times q}$ with

$$\begin{aligned} q_{ii} =&\frac{1}{10(i+1)}, \quad 1\leq i\leq p, \\ q_{ij} =&\frac{1}{30}-\frac{1}{30j+i}, \quad 1\leq i< j\leq p, \\ q_{ij} =&\frac{1}{30}-\frac{1}{30(i-j+1)+i}, \quad 1\leq j< i\leq p, \\ r_{ii} =&\frac{1}{10(p+i+1)}, \quad 1\leq i\leq q, \\ r_{ij} =&\frac{1}{30}-\frac{1}{30(p+j)+p+i}, \quad 1\leq i< j\leq q, \\ r_{ij} =&\frac{1}{30}-\frac{1}{30(i-j+1)+p+i}, \quad 1\leq j< i\leq q, \\ l_{ij} =&\frac{1}{30(p+i-j+1)+p+i}-\frac{1}{30}, \quad 1\leq i\leq q, 1\leq j\leq p, \\ u_{ij} =&\frac{1}{30(p+j)+i}-\frac{1}{30}, \quad 1\leq i\leq p, 1\leq j\leq q. \end{aligned}$$

It is obvious that *A* is an irreducible *M*-matrix.

Table 3 and Table 4 list $\rho (G^{-1}F)$, $\rho (\widetilde{G}^{-1}\widetilde{F})$, and $\rho (\hat{G}^{-1} \hat{F})$ with different *α* and Ω for Example 2, where in *P̃*, let $(\gamma_{1},\gamma_{2},\ldots ,\gamma_{n})^{T}=(0, \frac{1}{3},\ldots ,\frac{1}{3})^{T}$, $(\beta_{1},\beta_{1},\ldots , \beta_{n})^{T}=(0,0.003,\ldots ,0.003)^{T}$ and in *P̂*, let $( \gamma_{1},\gamma_{2},\ldots ,\gamma_{n})^{T}=(\frac{1}{3}, \frac{1}{3},\ldots ,\frac{1}{3})^{T}$, $(\beta_{1},\beta_{1},\ldots , \beta_{n})^{T}=(0.003,0.003,\ldots , [4]0.003)^{T}$.

**Table 3:** $\rho (G^{-1}F)$, $\rho (\widetilde{G}^{-1}\widetilde{F})$, and $\rho (\hat{G}^{-1}\hat{F})$ with $\alpha =0.1$ and $\omega_{i}=0.6$ for Example 2

*n*	*I*	*P̃*	*P̂*
5	0.46078	0.46040	**0.46038**
10	0.55689	0.55636	**0.55635**
15	0.65889	0.65833	**0.65832**
20	0.763952	0.763399	**0.763395**

**Table 4:** $\rho (G^{-1}F)$, $\rho (\widetilde{G}^{-1}\widetilde{F})$, and $\rho (\hat{G}^{-1}\hat{F})$ with $\alpha =0.2$ and $\omega_{i}=0.7$ for Example 2

*n*	*I*	*P̃*	*P̂*
5	0.37475	0.37400	**0.37398**
10	0.49391	0.49285	**0.49284**
15	0.62177	0.62063	**0.62062**
20	0.75501	0.75386	**0.75385**

From Tables 1, 2, 3, and 4 we can see that, for different choices of *α* and Ω, the inequalities

$$ \rho \bigl(\widehat{G}^{-1}\widehat{F} \bigr)\leq \rho \bigl( \widetilde{G}^{-1} \widetilde{F} \bigr)< 1 $$

hold, which verifies the theoretical result in Theorem 3.

## Conclusions

In this paper, we present a new preconditioner *P̂*, which provides the preconditioning effect on all the rows of *A*, to accelerate the convergence rate of the GAOR method to solve LCP (1) with an *M*-matrix *A*, and consider the preconditioned GAOR method (6). We prove that the original LCP (1) is equivalent to LCP (7), and show that the preconditioned GAOR method (6) is convergent for solving LCP (1). Then a comparison theorem on the preconditioned GAOR method (6) is obtained, which shows that the preconditioned GAOR method (6) improves the convergence rate of the preconditioned GAOR method in [13] for solving LCP (1). Together with the comparison result in [12], we know that the preconditioned GAOR method (6) improves considerably the convergence rate of the original GAOR method for solving LCP (1).
